# Inhibition of Rac1 activity by NSC23766 prevents cartilage endplate degeneration via Wnt/β‐catenin pathway

**DOI:** 10.1111/jcmm.15049

**Published:** 2020-02-10

**Authors:** Chao Jiang, Ze‐Ming Sun, Ding‐Chao Zhu, Qiang Guo, Jia‐Jing Xu, Jia‐Hao Lin, Ze‐Xin Chen, Yao‐Sen Wu

**Affiliations:** ^1^ Department of Orthopaedic surgery The Second Affiliated Hospital and Yuying Children's Hospital of Wenzhou Medical University Wenzhou China; ^2^ Zhejiang Provincial Key Laboratory of Orthopaedics Wenzhou China; ^3^ The Second School of Medicine Wenzhou Medical University Wenzhou China

**Keywords:** cartilage endplate, intervertebral disc, NSC23766, Rac1, Wnt/β‐catenin

## Abstract

Cartilage endplate (CEP) degeneration has been considered as one of important factors related to intervertebral disc degeneration (IVDD). Previous researches have showed that Rac1 played a pivotal role in chondrocyte differentiation. However, the effect of Rac1 during the process of CEP degeneration remains unclear. Herein, we explored the effect of Rac1 on CEP degeneration and elucidated the underlying molecular mechanism. We found expression of Rac1‐GTP increased in human‐degenerated CEP tissue and IL‐1β‐stimulated rat endplate chondrocytes (EPCs). Our study revealed that Rac1 inhibitor NSC23766 treatment promoted the expression of collagen II, aggrecan and Sox‐9, and decreased the expression of ADTAMTS5 and MMP13 in IL‐1β‐stimulated rat EPCs. Moreover, we also found that NSC23766 could suppress the activation of Wnt/β‐catenin pathway, suggesting that the beneficial effects of Rac1 inhibition in EPCs are mediated through the Wnt/β‐catenin signalling. Besides, puncture‐induced rats models showed that NSC23766 played a protective role on CEP and disc degeneration. Collectively, these findings demonstrated that Rac1 inhibition delayed the EPCs degeneration and its potential mechanism may be associated with Wnt/β‐catenin pathway regulation, which may help us better understand the association between Rac1 and CEP degeneration and provide a promising strategy for delaying the progression of IVDD.

## 1 INTRODUCTION

Intervertebral disc degeneration (IVDD) is considered as the crucial cause of low back pain, which influences approximately 80% of adults in different stages during their life‐time.^1^ Various influential factors including ageing, mechanical stress and inflammation are relevant to IVDD initiation and progression.^2^ Until recently, the exact aetiology and pathophysiology of IVDD have not been well elucidated.

Cartilage endplate (CEP) is an indispensable structure between the vertebral body and intervertebral disc, which was conducive to equalize loading between the disc and vertebrae and transport nutrients and metabolites through vertebral blood vessels.^3^ Minor damage to endplate could cause obvious structural changes in the adjacent intervertebral discs, altering the distribution of matrix compressive stress, and causing lower intradiscal pressure in the nucleus pulposus and greater compressive stresses in the annulus fibrosus.^4^ Wike et al have reported that CEP degeneration may be a possible contributor to the pathophysiology of disc degeneration, and its molecular mechanism involved the up‐regulation of matrix‐degrading enzymes and inflammatory cytokines.^5^ Moreover, Williams et al have demonstrated that endplate defect was significantly related to disc degeneration at every lumbar disc level through a large population‐based study.^6^ Therefore, CEP degeneration may be a pivotal initiating factor in disc degeneration pathogenesis. Nevertheless, the specific molecular mechanism of CEP degeneration is still not clear.

Rac1 is one of small GTPases of Rho family, which plays vital role in chondrocyte differentiation and cell proliferation.^7^, ^8^ Previous studies have identified the effect of aberrant activation of Rac1 in promoting chondrocyte hypertrophy, apoptosis and mineralization.^9^, ^10^, ^11^ And the effect of Rac1 on the regulation of chondrocyte differentiation was proved by genetically modified mice.^12^ David et al have reported that Rac1 signalling in chondrocytes stimulated by fibronectin fragment resulted in increased MMP13, which was involved in the cartilage matrix destruction.^13^ Recently, Ouyang et al have demonstrated that activation of Rac1 could promote cartilage destruction and accelerate osteoarthritis development, while Rac1 inhibition prevented cartilage against osteoarthritis in vivo.^14^ Therefore, it is logical to explore whether Rac1 is also involved in the pathological process of CEP degeneration during IVDD, and this deserves further study on the relationship between Rac1 and CEP degeneration and its underlying molecular mechanism.

Recently, Wnt/β‐catenin pathway has been demonstrated to play critical role in cartilage long‐term function, which implicated in the regulation of cell proliferation, maintenance of phenotypic characteristics and chondrocyte differentiation.^15^, ^16^, ^17^ Wnt/β‐catenin pathway has been recognized as one of the master regulators involved in the IVDD development.^18^, ^19^ In addition, Takahito et al have suggested Wnt/β‐catenin signalling was a powerful stimulator of chondrocyte matrix catabolic action, leading to the degradation of cartilage matrix.^16^ Moreover, Zhang et al have shown that intermittent cyclic mechanical tension‐induced cartilaginous endplate degeneration may attribute to Wnt/β‐catenin signalling to some extent.^20^ In addition, Rac1 was reported to control β‐catenin phosphorylation and nuclear localization, providing novel target for therapeutic intervention of Wnt/β‐catenin pathway.^21^ Herein, the intention of present study was to investigate the effect of Rac1 in the degeneration of cartilaginous endplate and potential relationship with Wnt/β‐catenin pathway through IL‐1β‐induced endplate chondrocytes (EPCs) in vitro and rat annulus needle puncture models of IVDD in vivo.^22^, ^23^, ^24^ We suggested that Rac1 inhibition could be a promising therapeutic strategy for CEP degeneration and even IVDD.

## 2 MATERIALS AND METHODS

### 2.1 Ethics statement

All experimental procedures and the animal use and care protocols were conducted on the basis of the Animal Care and Use Committee of Wenzhou Medical University. Human CEP tissue collection and experiments that involved human CEP were approved by the Second Affiliated Hospital and Yuying Children's Hospital of Wenzhou Medical University Ethics Committee (L‐2018‐46) and followed the guidelines of the Helsinki Declaration.^25^

### 2.2 Reagents and antibodies

Recombinant rat IL‐1β was obtained from Peprotech. NSC23766 was obtained from MedChemExpress. XAV‐939 and SKL2001 were purchased from Meilunbio. Antibodies against Rac1‐GTP, Rac1‐Total and Sox‐9 were purchased from Cell Signaling Technology. Antibodies against β‐actin and normal rabbit IgG were purchased from Santa Cruz Biotechnology. Antibodies against aggrecan, collagen II, ADAMTS5 and MMP13 were purchased from Abcam.

### 2.3 Human endplate chondrocytes culture

The normal human CEP tissue (Pfirrmann grades I‐II, n = 8) was obtained from patients undergoing treatment of lumbar fracture with lumbar internal fixation. The degenerated human CEP tissues (Pfirrmann grades IV‐V, n = 10) from IVDD patients. To isolated primary human endplate chondrocytes (EPCs), CEP tissue was cut up into 1 mm^3^ and treated with 0.2% type II collagenase for 4 hours at 37℃. After washing with phosphate‐buffered saline (PBS), human EPCs were added to the 10‐cm culture plates with appropriate density, and cells were cultured in DMEM/ F12 supplemented with 10% foetal bovine serum (FBS) and antibiotics (1% streptomycin/penicillin) incubate in 5% CO_2_ and 37°C. The complete medium was changed every other day, and the first three passages of chondrocytes were used for the experiments.

### 2.4 Primary rat endplate chondrocytes culture

Cartilaginous endplate tissues were extracted from the tails of 2‐week‐old rats (Sprague Dawley rats, n = 10, five males and five females) under a dissecting microscope. The cartilage tissues were digested with 0.2% type II collagenase for 4 hours at 37°C. Next, the rat EPCs were added to the 10‐cm culture plates with appropriate density and cultured in DMEM/F12 supplemented with 10% FBS and antibiotics (1% streptomycin/penicillin) and maintained in 5% CO_2_ and 37°C. The complete medium was changed every other day, and the first three passages of chondrocytes were used for the experiments.

### 2.5 Western blotting

The proteins of EPCs were extracted using ice‐cold RIPA with 1 mmol/L phenylmethanesulphonyl fluoride (PMSF), and the protein concentration of samples was measured using the bicinchoninic acid method through BCA protein assay kit (Beyotime). The proteins of EPCs were separated by 8%‐12% (*w*/*v*) sodium dodecyl sulphate‐polyacrylamide gel electrophoresis (SDS‐PAGE) and were transferred to a polyvinylidene difluoride membrane (Millipore). After blocking with 5% nonfat milk, the bands were subsequently incubated with primary antibodies specific to Rac1‐Total (1:1000), Rac1‐GTP (1:1000), aggrecan (1:1000), collagen II (1:1000), Sox‐9 (1:1000), ADAMTS5 (1:1000), MMP13 (1:1000), β‐catenin (1:1000), SFRP1 (1:1000), lamin b (1:1000) and β‐actin (1:1000) overnight at 4°C, followed by incubation with the appropriate secondary antibodies. Last, the intensity of the bands was visualized and analysed using Image Lab 3.0 software (Bio‐Rad).

### 2.6 Immunofluorescence

For immunofluorescence, EPCs were added to six‐well glass plates and cultured with serum‐starved medium overnight. Next, the cells were treated with 10 ng/mL IL‐1β or were co‐treated with 10 ng/mL IL‐1β and 50 μmol/L NSC23766 or were co‐treated with 10 ng/mL IL‐1β and 10 μmol/L XAV‐939 or were co‐treated with 10 ng/mL IL‐1β, 50 μmol/L NSC23766 and 40 μmol/L SKL2001 for 4 hours in medium. After washing with PBS, the cells were fixed in 4% paraformaldehyde and incubated with 0.5% Triton X‐100 for 15 minutes. Then, the cells were blocked with 10% bovine serum albumin for 1 hour at 37°C, and incubated with primary antibodies against Sox‐9 and collagen II overnight at 4°C, followed by incubation with appropriate secondary antibodies for 1 hour at room temperature and stained for 5 minutes with DAPI. Images were captured with a fluorescence microscope (Olympus Inc).

### 2.7 Quantitative RT‐PCR

Total RNA of EPCs was extracted using TRIzol (Invitrogen) according to the manufacturer's instructions. cDNA was synthesized using 1 μg RNA through the One Step RT‐PCR Kit (TaKaRa). Quantitative real‐time PCR was analysed using the iQTM SYBR Green Supermix PCR kit through the iCycler apparatus system (Bio‐Rad). All primer sequences were listed as follows: for aggrecan, Forward: 5′‐AAGTGCTATGCTGGCTGGTT‐3′, Reverse: 5′‐GGTCTGGTTGGGGTAGAGGT‐5′; for collagen II, Forward: 5′‐CTCAAGTCGCTGAACAACCA‐3′, Reverse: 5′‐GTCTCCGCTCTTCCACTCTG‐3′; for MMP13, Forward: 5′‐CCAGAACTTCCCAACCAT‐3′, Reverse: 5′‐ACCCTCCATAATGTCATACC‐3′; for ADAMTS5, Forward: 5′‐GGGAATAAGTACTGGGCTGTTCAG‐3′, Reverse: 5′‐CCTCAGAAAGAGCAGCATCGATATG‐3′; for Axin2, Forward: 5′‐ GAACCTGAAGGATCGCAAAA3′, Reverse: 5′‐ GGTTTCAGCTGCTTGGAGAC‐3′; for LEF‐2, Forward: 5′‐ CTGAGCGAGTTGTGTACCG‐3′, Reverse: 5′‐AGTTCCTCCGAAAATGTAGGG‐3′; for β‐actin, Forward: 5′‐TCTCCTCTGACTTCAACAGCGAC‐3′, Reverse: 5′‐CCCTGTTGCTGTAGCCAAATTC‐3′.

### 2.8 Animal model

Adult Sprague Dawley rats (200‐250 g, male, n = 18) were anaesthetized with 2% (*w*/*v*) pentobarbital (40 mg/kg) and randomly divided into three groups: control group (n = 8 each group), IVDD group and IVDD + NSC23766 group. The coccygeal intervertebral space Co7‐8 was located by palpation and confirmed with an X‐ray radiograph which was selected for the further study. After the rat tail skin was sterilized with iodophor, needles (26G) were applied to perpendicularly puncture the whole layer of annulus fibrosus though the tail skin and needles were maintained for 1min in the disc. After surgery, the rats in IVDD + NSC23766 group were intraperitoneal injected with NSC23766 (50 mg/kg) while other rats were administered with the same amount of saline every 2 days until the rats were sacrificed.^14^

### 2.9 Histological assessment

At 8 weeks after surgery, all rats were killed and disc specimens were fixed in 4% paraformaldehyde and decalcified, then the specimens were dehydrated and embedded in paraffin. The tissues were cut into 5‐μm sections. Sections were stained with safranin O‐fast green (SO) due to the protocol. Images were visualized and captured under a microscope.

### 2.10 Immunohistochemical analysis

The sections(5 μm) were deparaffinized in xylene and rehydrated in ethanol, and endogenous peroxidase was blocked by 3% (*v*/*v*) H_2_O_2_ for 15 minutes. The sections were incubated with 0.4% pepsin in 5mM HCl for 20 minutes at 37℃ for antigen retrieval, followed with incubation of 5% bovine serum albumin for 30 minutes. After incubation with primary antibody overnight at 4°C, the sections were finally incubated with HRP conjugated secondary antibody. Images were visualized and captured under a microscope. The rate of positive cells during each section was analysed by a separate group of experienced histology researchers who were blinded to the experimental groups.

### 2.11 Magnetic resonance imaging

At 8 weeks after surgery, magnetic resonance imaging (MRI) of the coccyx was performed to evaluate the IVDD. The IVDD signal and structural changes in sagittal T2‐weighted images using a 3.0‐T clinical magnet (Philips Intera Achieva 3.0MR) were assessed through MR imaging. The parameters of T2‐weighted in the sagittal plane were as following: fast spin echo sequence with time to repetition (TR) of 5400ms and time to echo (TE) of 920 ms; 320 (h) 9256 (v) matrix; field of view 260; and four excitations. The section thickness was 2 mm with a 0‐mm gap. The degree of IVDD during MRIs images was evaluated by another three orthopaedic researchers in a blinded manner according to the Pfirrmann grade.^26^

### 2.12 Statistical analysis

The results are expressed as the mean ± SD Statistical analysis was performed using SPSS statistical software program 20.0. Data were analysed by one‐way analysis of variance (ANOVA) followed by the Tukey's test for comparison between the two groups. *P* < .05 was considered statistically significant.

## 3 RESULTS

### 3.1 Aberrant activation of Rac1 in degenerated human endplate chondrocytes and IL‐1β‐stimulated rats endplate chondrocytes

**Figure 1:**
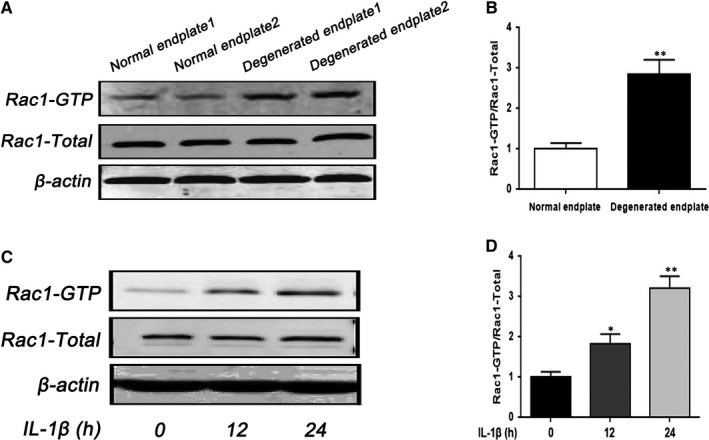
The expression of Rac1 increased in human‐degenerated CEP and IL‐1β‐stimulated rat EPCs. A, B, The expression of Rac1 from EPCs of normal and IVDD patients was analysed by Western blot and its quantification. C, D, The effect of different durations of treatment with IL‐1β (10 ng/mL) on rat EPCs was analysed by Western blot and its quantification. All experiments were performed in duplicates, and data are reported as the mean ± SD. (n = 6). *P < .05, **P < .01

In order to illuminate the relationship between CEP degeneration and Rac1, we compared the expression of Rac1 during normal and degenerated human EPCs and evaluated the expression of Rac1 in IL‐1β‐induced rats EPCs by Western blotting analysis. According to the results, the level of Rac1‐GTP was markedly increased in degenerated EPCs compared with normal chondrocytes in protein level (Figure 1A,B). And Western blotting analysis and its quantification revealed that IL‐1β (10 ng/mL) promoted the protein expression of Rac1‐GTP in a time‐dependent manner in rat EPCs (Figure 1C,D).^27^ Thus, we conclude that Rac1 might be associated with CEP degeneration.

### 3.2 NSC23766 alleviated IL‐1β‐induced the extracellular matrix molecules (ECM) degradation and Sox‐9 expression in rat EPCs

**Figure 2:**
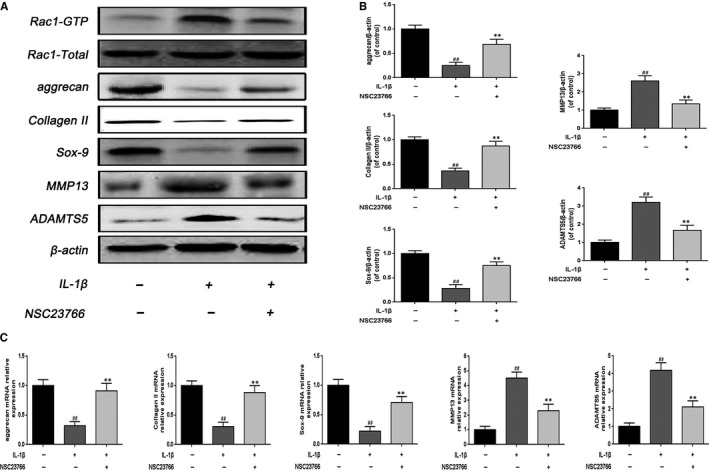
Effect of Rac1 inhibitor NSC23766 on the expression of degeneration‐related genes and Sox‐9 in rats EPCs. A, B, The effect of NSC23766 (50 μmol/L) on the level of aggrecan, collagen II, Sox‐9, MMP13 and ADAMTS5 was analysed by Western blot and its quantification. C, The mRNA expression of aggrecan, collagen II, Sox‐9, MMP13 and ADAMTS5 was measured by real‐time PCR. All experiments were performed in duplicates, and data are reported as the mean ± SD. (n = 6). ##*P* < .01 compared with the control group. **P < .01 compared with the IL‐1β group

**Figure 3:**
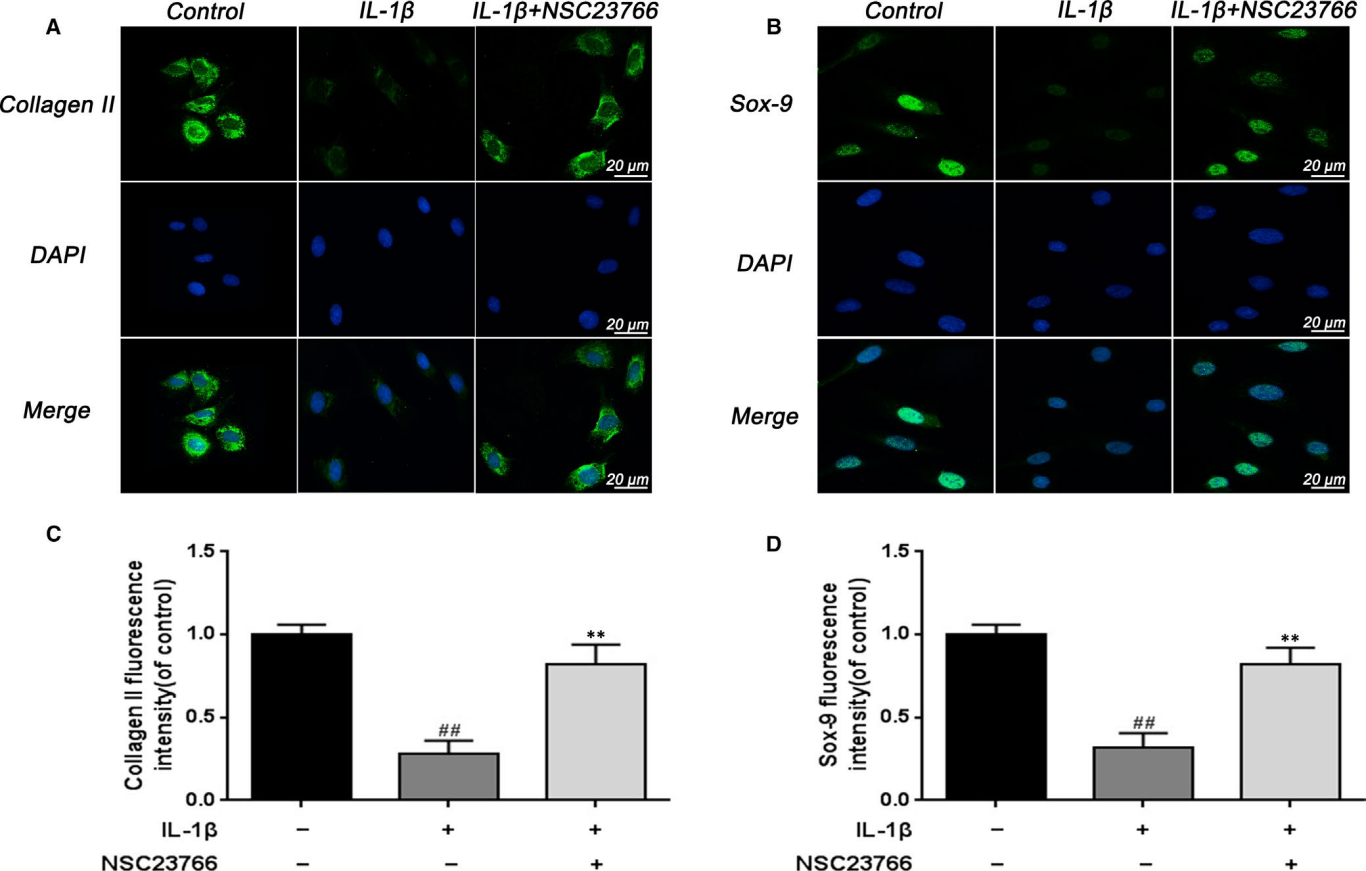
Effect of Rac1 inhibitor NSC23766 on the fluorescence expression of collagen II and Sox‐9 in rats EPCs. A, Immunofluorescence of collagen II was observed by fluorescence microscope (OLYMPUS). Scale bar: 20 μm. B, Immunofluorescence of Sox‐9 was observed by fluorescence microscope (OLYMPUS). Scale bar: 20 μm. C, Quantitation of immunofluorescence staining of collagen II. D, Quantitative analysis of immunofluorescence staining of Sox‐9. All experiments were performed in duplicates, and data are reported as the mean ± SD. (n = 6). ^##^ *P* < .01 compared with the control group. **P < .01 compared with the IL‐1β group

To investigate the Rac1 inhibition function on IL‐1β‐induced ECM degradation, we tested the effect of Rac1 inhibitor NSC23766 on the expression of aggrecan, collagen II, MMP13 and ADAMTS5 through Western blot and real‐time PCR. As shown in Figure 2A,B, IL‐1β decreased the expression of aggrecan and collagen II, and up‐regulated the expression of MMP13 and ADAMTS5, whereas Rac1 inhibitor NSC23766 (50 μmol/L) could reverse its effect on protein level.^28^ In addition, the result of real‐time PCR analysis was basically consistent with Western blot analysis (Figure 2C). Notably, immunofluorescence staining of collagen II further confirmed the effect of NSC23766 (Figure 3A,C).

It is generally accepted that Sox‐9 plays a vital role in the regulation of aggrecan and collagen II, we examined the effect of NSC23766 on Sox‐9 expression. As shown in Figure 2A,B,C, NSC23766 significantly activated Sox‐9 expression following IL‐1β stimulation. And the result of immunofluorescence staining of Sox‐9 was in accordance with the protein and mRNA results (Figure 3B,D).

Altogether, these results indicate the protective effect of NSC23766 on ECM degradation in rat EPCs.

### 3.3 NSC23766 inhibited the Wnt/β‐catenin pathway activation in IL‐1β‐induced rat EPCs

**Figure 4:**
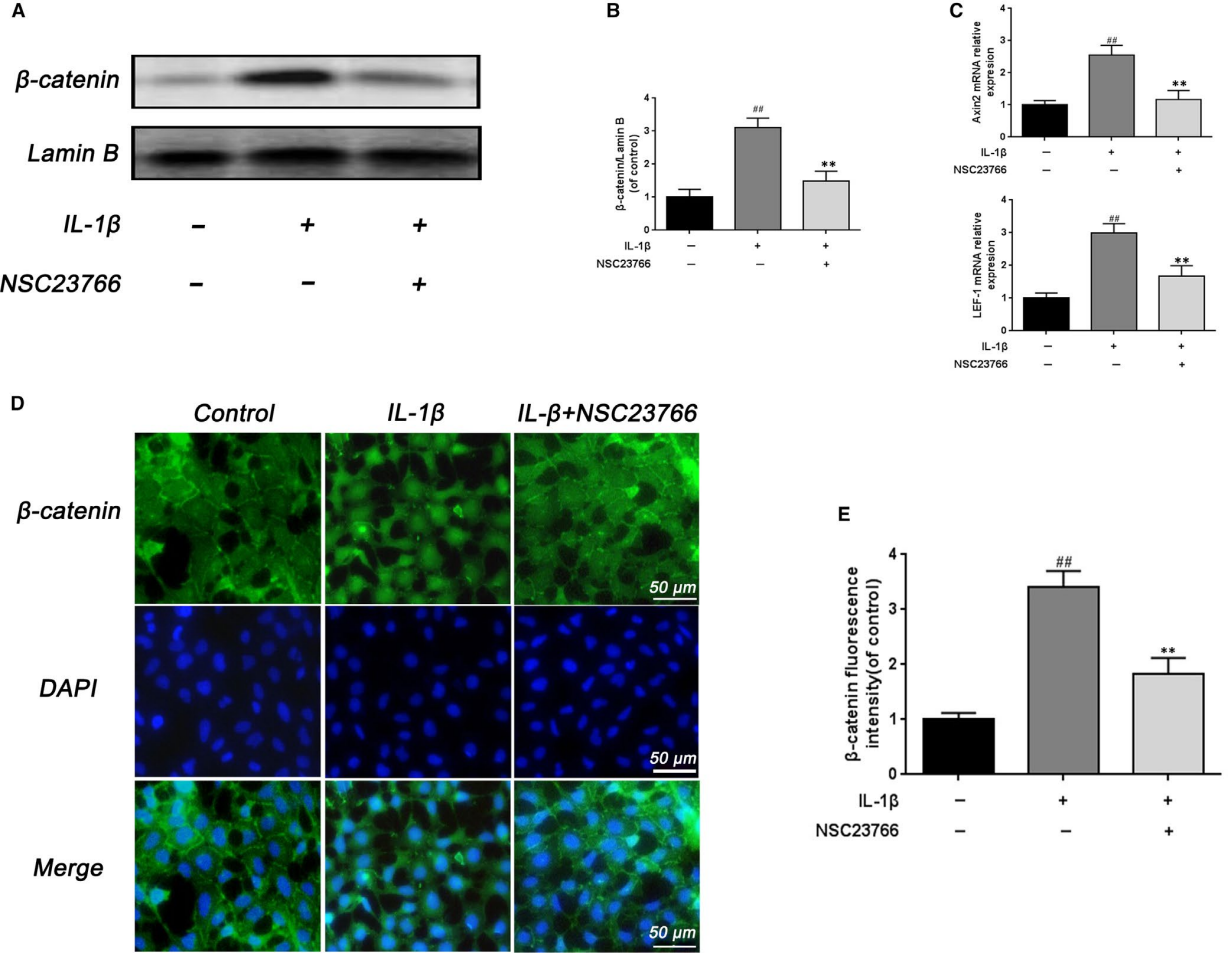
The effect of Rac1 inhibitor on Wnt/β‐catenin signalling in rat EPCs. A, B, The effect of NSC23766 on the level of β‐catenin was analysed by Western blot and its quantification. C, The effect of NSC23766 on the level of Axin2 and LEF‐1 was analysed by real‐time PCR. D, Immunofluorescence of β‐catenin was observed by fluorescence microscope (OLYMPUS). Scale bar: 50 μm. E, Quantitative analysis of immunofluorescence staining of β‐catenin in the nuclei of rat EPCs. All experiments were performed in duplicates, and data are reported as the mean ± SD. (n = 6). ^##^ *P* < .01 compared with the control group. **P < .01 compared with the IL‐1β group

To further clarify the potential mechanism of Rac1 on CEP, we investigated the effect of Rac1 inhibition on the Wnt/β‐catenin pathway in rat EPCs. We isolated the nuclear protein from EPCs and compared the β‐catenin expression in the nucleus. After IL‐1β stimulation, the β‐catenin level in the nucleus was up‐regulated whereas NSC23766 treatment inhibited this response (Figure 4A,B). Moreover, the Wnt downstream signalling Axin2 and LEF‐1 were used to better identify the relationship between the Rac1 and Wnt/β‐catenin pathway. mRNA level analysis of Axin2 and LEF‐1 showed that IL‐1β stimulation markedly activated the Axin2 and LEF‐1, which could be inhibited by NSC23766 (Figure 4C). In addition, β‐catenin immunofluorescence was performed to evaluate the translocation of β‐catenin from the cytoplasm to nucleus in rat EPCs. During control EPCs, β‐catenin mainly localized in the cytoplasm, whereas β‐catenin markedly translocated to the nucleus after stimulation of IL‐1β. NSC23766 treatment obviously inhibited IL‐1β‐induced nuclear translocation of β‐catenin (Figure 4D), which were consistent with the results of Western blot. The result of β‐catenin fluorescence intensity confirmed the effect of NSC23766 (Figure 4E).

**Figure 5:**
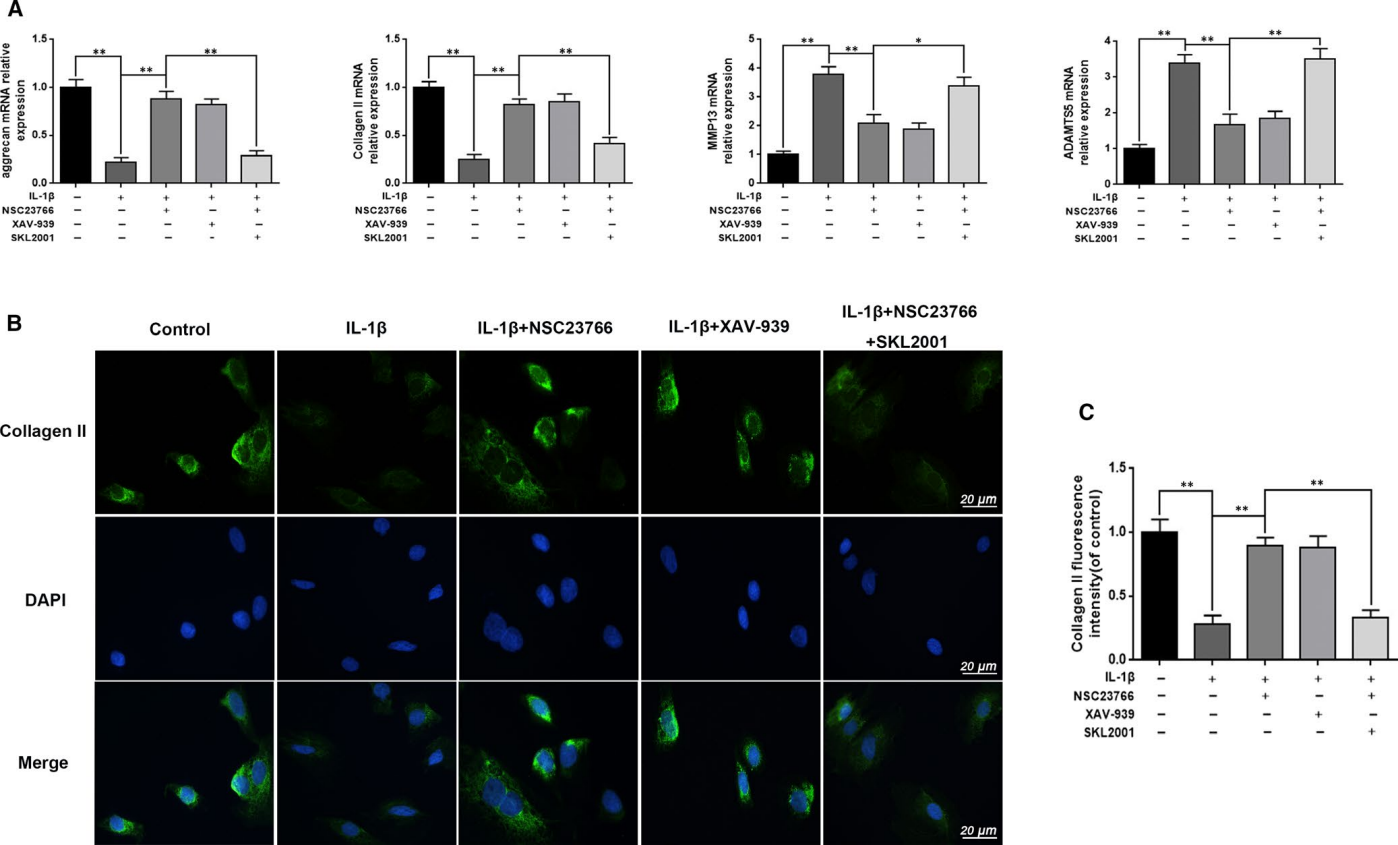
Inhibition of Wnt/β‐catenin pathway suppresses the effects of NSC23766. A, The mRNA expression of aggrecan, collagen II, Sox‐9, MMP13 and ADAMTS5 was measured by real‐time PCR. B, Immunofluorescence of collagen II was observed by fluorescence microscope (OLYMPUS). Scale bar: 20 μm. C, Quantitation of immunofluorescence staining of collagen II. All experiments were performed in duplicates, and data are reported as the mean ± SD. (n = 6). *P < .05, **P < .01

Moreover, Wnt/b‐catenin signalling inhibitor and activator were introduced to further confirm the interaction between the Rac1 and Wnt/β‐catenin pathway in rat EPCs. As shown in Figure 5A, real‐time PCR analysis showed that Wnt/b‐catenin signalling inhibitor XAV‐939 (10 μmol/L) could mimic the effects of NCS23766,^29^ which increased the expression of aggrecan and collagen II and decreased the expression of MMP13 and ADAMTS5 after IL‐1β stimulation, while Wnt/b‐catenin signalling activator SKL2001 (40 µmol/L) could rescue the effects of NCS23766.^30^ And the result of immunofluorescence staining of collagen II was in accordance with the mRNA results (Figure 5B,C).

These results prove the inhibitory effect of NSC23766 on Wnt/β‐catenin pathway in rat EPCs.

### 3.4 **NSC23766 ameliorated CEP and disc degeneration in a puncture**‐**induced rat model**

**Figure 6:**
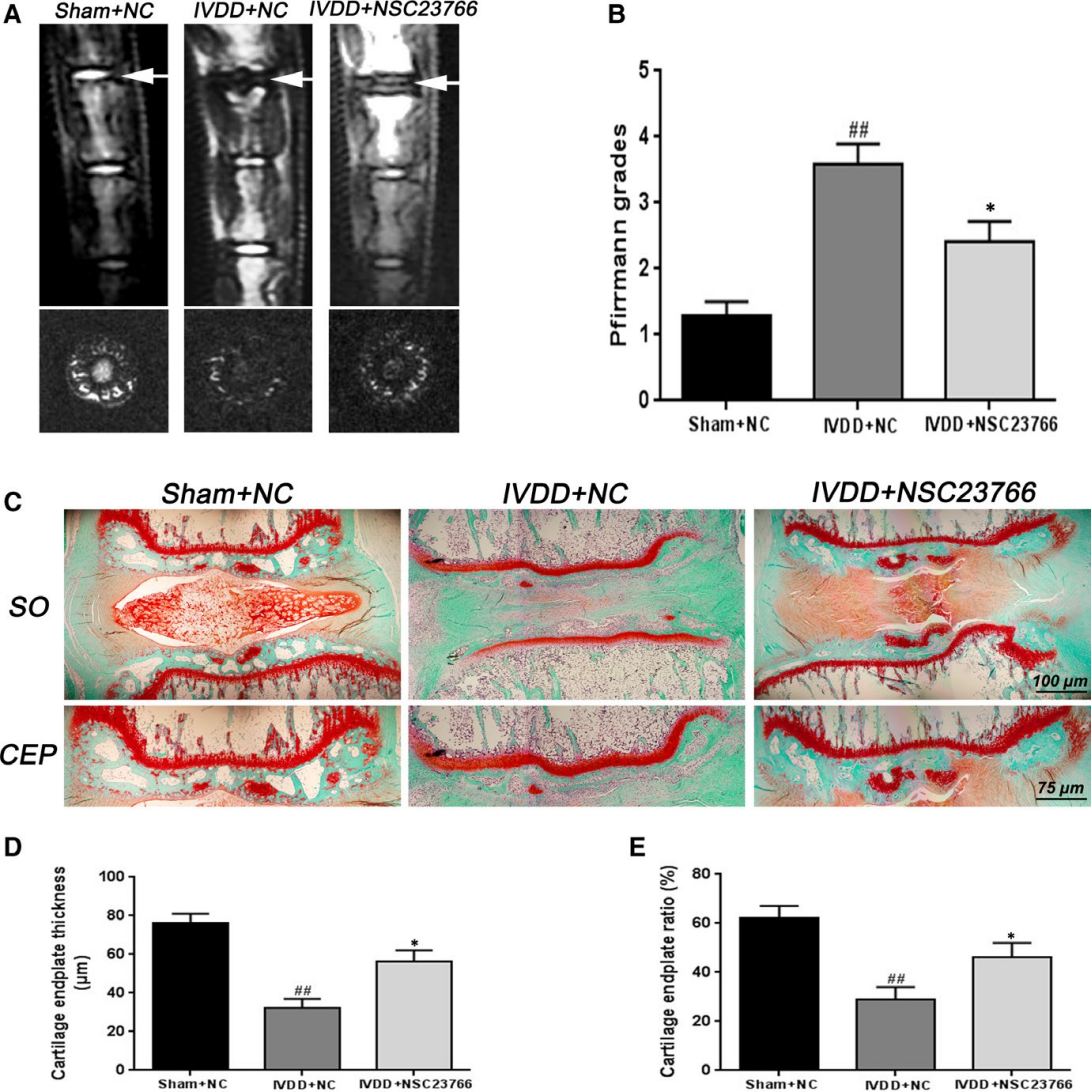
Rac1 inhibitor NSC23766 prevents CEP degeneration and disc degeneration in vivo. A, T2‐weighted MRI of a rat tail with a needle‐punctured disc at 8 wk postoperatively (the white arrow). B, The Pfirrmann MRI grade scores were acquired from three groups at week 8. C, Representative SO staining of disc and CEP in different group. Scale bar: 100 μm. D, E, The quantitation of CEP thickness and chondrocyte/CEP area in different group. All experiments were performed in duplicates, and data are reported as the mean ± SD. (n = 6). ^##^ *P* < .01 compared with the Sham + NC group. *P < .05 compared with the IVDD + NC group

Our studies also evaluated the protective effects of NSC23766 on IVDD development in vivo, and rats IVDD model was established through disc puncture surgery. Magnetic resonance imaging (MRI) was taken at 8 weeks to evaluate the degree of disc degeneration in rats. From MRI results, we found higher T2‐weight signal intensity of the intervertebral disc in NSC23766‐treated group than that in IVDD group (Figure 6A). Besides, the Pfirrmann MRI grade scores, indicating the degree of disc degeneration, were also significantly lower in the NSC23766‐treated group compared with the saline group (Figure 6B). SO staining was applied for further histological analysis of IVDD. SO staining of disc showed that the structure of CEP disappeared (Figure 6C), the thickness of CEP became thinner and the proportion of EPCs in CEP areas gradually reduced in IVDD group, while NSC23766 treatment delayed these histopathological changes (Figure 6D,E). Interestingly, compare to the IVDD group, nucleus pulposus tissues were also better preserved after NSC23766 treatment (Figure 6C), suggesting that inhibition of Rac1 activity may also be beneficial for nucleus pulposus tissues.

**Figure 7:**
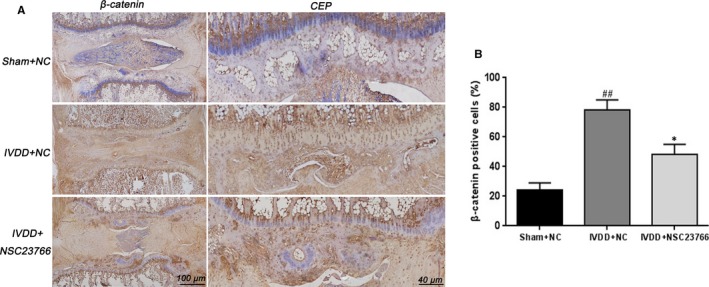
Rac1 inhibitor NSC23766 suppresses β‐catenin activation during disc and CEP in vivo. A, Immunohistochemical staining of β‐catenin expression in the disc and CEP samples of each group. Scale bar: 100 μm. B, Relative positive cells of β‐catenin in CEP sample were quantified by image pro plus. All experiments were performed in duplicates, and data are reported as the mean ± SD. (n = 6). ^##^ *P* < .01 compared with the Sham + NC group. *P < .05 compared with the IVDD + NC group

Based on the vitro study results, the effect of NSC23766 on the Wnt/β‐catenin signalling in vivo was further verify. Consistent with the vitro findings, immunohistochemical staining and its corresponding quantification demonstrated that NSC23766 could inhibit the β‐catenin expression (Figure 7A,B), and promote the Sox‐9 activation in CEP and disc tissue (Figures S1A,B). Collectively, these results provide a strong evidence regarding the protective effect of Rac1 inhibition on CEP and disc degeneration in puncture‐induced rat model.

## 4 DISCUSSION

This study was designed to clarify the effect of Rac1 activity in CEP degeneration and disc degeneration and seek for the effective strategy for prevention of IVDD development. The present work, for the first time, illustrated the aberrant activation of Rac1 in CEP degeneration, and Rac1 inhibitor NSC23766 protected the disc from IVDD through CEP regulation via Wnt/β‐catenin pathway.

The Rho family of small GTPases has been proved to play critical role in numerous aspects of cell biology, including cell proliferation, apoptosis, migration and invasion.^8^, ^31^ Recently, the relationship between Rho family and chondrocytes has been thoroughly studied.^32^, ^33^ Three major types of molecules were involved in the Rho family: Cdc42, Rac1 and RhoA. Rac1, Cdc42 and RhoA are all expressed in articular and growth plate chondrocytes, but the functions differ among them. Rac1 and Cdc42 promote chondrocyte differentiation while RhoA exerts an antagonistic effect on chondrocyte development.^10^, ^12^ Recent studies have identified that regulation of Rac1 activity was necessary for normal cartilage development which might be potently therapeutic target to associated diseases such as osteoarthritis.^32^, ^33^ Work from Beier et al have suggested that Rac1 activity was required for the chondrocyte hypertrophy marker type X collagen, indicating the effective regulation of cartilage development.^10^ Nevertheless, the role of Rac1 activity in CEP has remained unclear and needs further exploration. We found that the level of Rac1 was elevated not only in human‐degenerated CEP tissues and Rac1 expression substantially increased by the proinflammatory factor IL‐1β in a time‐dependent manner comparing to the expression levels of Rac1 in the untreated control group in rat EPCs. In addition, Rac1 inhibitor NSC23766 obviously up‐regulated the level of anabolic genes, collagen II and aggrecan, and lowered the expression of catabolic genes, MMP13 and ADAMTS5 following IL‐1β stimulation. Sox‐9 is a major transcription factor of chondrocyte differentiation, which is closely related with to the regulation of collagen II and aggrecan. Present study showed that the expression of Sox‐9 was markedly decreased in IL‐1β‐stimulated EPCs cells, which could be reversed by Rac1 inhibition. This is a novel finding and has not been previously reported in the field of CEP and disc degeneration, which provided a new insight of IVDD treatment.

Wnt/β‐catenin signalling has been proved as a crucial factor in regulating several biological processes such as cell fate determination during embryogenesis, cell proliferation, differentiation and apoptosis.^34^, ^35^, ^36^ Thus, aberrations of Wnt/β‐catenin pathway are often related to defects in cellular differentiation. Previous researches have demonstrated that the Wnt/β‐catenin pathway plays a pivotal role in chondrocyte proliferation and hypertrophic differentiation,^17^, ^37^ while inhibition of Wnt signalling could promote the expression of chondrogenic transcription factor Sox‐9 and cartilage‐related gene collagen II.^38^ Moreover, Akihiko et al have proved that activation of Wnt/β‐catenin signalling could promote cellular senescence and may lead to an increased breakdown of the matrix, thereby promoting disc degeneration.^18^ Recently, Lei et al have illustrated that Wnt/β‐catenin pathway engaged in the CEP homeostasis and degeneration, further revealing the intracellular regulatory mechanism of CEP degeneration.^39^ Therefore, inhibition of Wnt/β‐catenin signalling may be a potential strategy for preventing CEP degeneration.

The relationship between the Rac1 and Wnt/β‐catenin signalling has been thoroughly studied to date. Wu et al have demonstrated that Rac1 activation could cause the elevated catenin level and nuclear translocation and contribute to aberrant activation of canonical Wnt signalling in cancer cells, indicating the important roles of Rac1 in β‐catenin nuclear localization and β‐catenin‐dependent transcriptional activation.^21^ Moreover, Jamieson et al have proved that Rac1 enhanced the interaction between β‐catenin and LEF‐1, further identifying the role of Rac1 in augmenting transactivation of Wnt target genes.^40^ Buongiorno et al have demonstrated that Rac1 and the Rac1‐specific activator Tiam1 were components of transcriptionally active beta‐catenin/TCF complexes at Wnt‐responsive promoters, revealing a novel functional mechanism underlying the cross‐talk between Rac1 and the canonical Wnt signalling pathway in colorectal cancer cells.^41^ Wan et al have discovered that Rac1 and Cdc42 are critical regulator of β‐catenin signalling in shear stress‐induced osteoblasts.^42^ In addition, Ouyang et al have revealed that Rac1 activation could directly interact with β‐catenin in modulating pathological changes in chondrocytes.^14^ In the current study, Rac1 inhibition by NSC23766 suppressed the nuclear translocation of β‐catenin and decreased the expression of β‐catenin induced by IL‐1β, which was accordance with the results of Ouyang et al.^14^

Axin2 and LEF‐1 are one of the major downstream factors of Wnt/β‐catenin signalling. Our data showed that mRNA expression of Axin2 and LEF‐1 was markedly reduced in NSC23766 treatment group than the IL‐1β‐stimulated group. Taken together, these results indicate that the protective role of Rac1 inhibition in rat EPCs may attribute to the regulation of Wnt/β‐catenin pathway.

Based on the beneficial effects of NSC23766 in rat EPCs, rat annulus needle puncture models of IVDD were introduced to evaluate the therapeutic value of Rac1 inhibition in in vivo study. Interestingly, in addition to endplate cartilage, the nucleus pulposus was also better preserved in the NSC23766‐treated group than in the IVDD group, indicating that NSC23766 may contribute to the prevention of IVDD also through regulating nucleus pulposus. In addition, our study showed that NSC23766 reduced the β‐catenin expression both in endplate cartilage and nucleus pulposus, suggesting that NSC23766 alleviated the development of IVDD by targeting Wnt/β‐catenin signalling.

In conclusion, our research revealed that expression of Rac1 was increased in human‐degenerated EPCs in vivo and IL‐1β‐stimulated rat EPCs in vitro. Rac1 inhibitor NSC23766 could suppress the degradation of EPCs through the regulation of Wnt/β‐catenin signalling. Moreover, our in vivo experiments demonstrated that NSC23766 may ameliorate the development of CEP and disc degeneration in IVDD model. Thus, inhibition of Rac1 could be a effective therapeutic strategy for IVDD treatment.

## CONFLICTS OF INTEREST

The authors declare no conflict of interests.

## AUTHORS’ CONTRIBUTION

YSW, CJ and ZMS contributed to conceptualization; DCZ contributed to methodology; QG contributed to software; JJX and JHL contributed to validation; ZXC and ZMS contributed to formal analysis; DCZ and JHL contributed to data curation; CJ and ZMS contributed to writing‐original draft preparation; YSW contributed to writing‐review and editing; ZMS and ZXC contributed to supervision; QG, JJX and ZXC contributed to project administration; YSW contributed to funding acquisition.

## Data Availability

All original data used in this work will be made available upon reguest.
