# Supplementary figures and images for: Inhibition of Rac1 activity by NSC23766 prevents cartilage endplate degeneration via Wnt/β‐catenin pathway
Source: J Cell Mol Med. 2020 Feb 10;24(6):3582–92. doi: 10.1111/jcmm.15049 (PMC7131937; doi:10.1111/jcmm.15049)

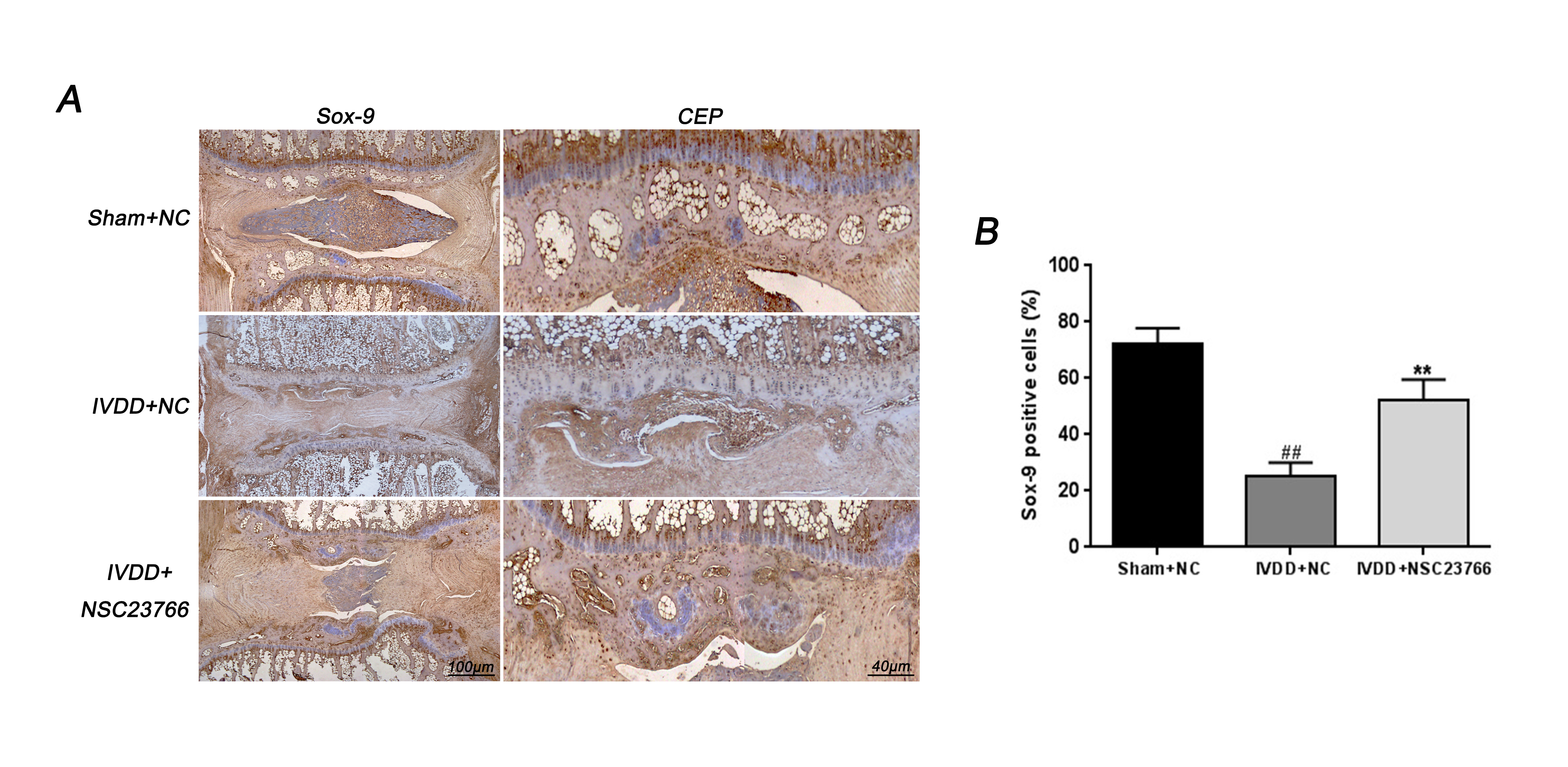

Supplement: Supplementary file 1 [file JCMM-24-3582-s001.tif]
